# Nitrogen acquisition strategy shifts with tree age depending on root functional traits and soil properties in *Larix principis-rupprechtii* plantations

**DOI:** 10.3389/fpls.2024.1358367

**Published:** 2024-03-12

**Authors:** Qianyuan Liu, Yaxuan Chen, Yanmei Chen

**Affiliations:** ^1^ Hebei Key Laboratory of Environmental Change and Ecological Construction, School of Geographical Sciences, Hebei Normal University, Shijiazhuang, China; ^2^ Geography Postdoctoral Research Station at Hebei Normal University, Shijiazhuang, China

**Keywords:** nitrogen uptake, root economics space, tree age, root traits, mycorrhizal colonization

## Abstract

**Introduction:**

Variation in plant nitrogen uptake rate and substrate preference is complicated due to the combined influence of abiotic and biotic factors. For the same species of tree across different ages, the interactions between root structural traits, nitrogen uptake rate, and soil environment have not been fully characterized, a situation that constrains our understanding of underground resource strategies employed by trees at different ages.

**Methods:**

In the present study, we examined the nitrogen uptake rate, mycorrhiza, morphology, architecture, and chemistry of the roots of *Larix principis-rupprechtii* in a chronosequence (aged 18, 27, 37, 46, and 57 years) in the Saihanba Mechanical Forest Farm in Northern China.

**Results:**

*L. principis-rupprechtii* preferred to absorb ammonium, followed in order by glycine and nitrate. The ammonium uptake rate of *L. principis-rupprechtii* significantly decreased (aged 18–37 years) and then increased (aged 46–57 years) with tree age. The glycine, nitrate, and total nitrogen uptake rates decreased with tree age. The root resource acquisition strategy appeared to shift from an acquisitive strategy to a conservative strategy associated with increasing tree age.

**Discussion:**

Along the root-mycorrhizal collaboration gradient, the younger *L. principis-rupprechtii* relied more on their own root morphology and physiology to acquire resources, adopting a “do it yourself” strategy comprising increasing the specific root length, the specific root area, and the N uptake rate (nitrate and glycine). Conversely, older trees depended more on mycorrhizal partners to acquire nitrogen resources, an “outsourcing” strategy. The results contribute to our understanding of underground resource-use strategies of plants and the nitrogen cycle in forest ecosystems according to stand age.

## 1 Introduction

Nitrogen (N) is one of the most important and limiting elements in forest ecosystems (LeBauer and Treseder, 2008). Plants utilize nitrogen in various forms, including ammonium (NH_4_^+^–N), nitrate (NO_3_^−^–N), and organic nitrogen, and the absorbed nitrogen contributes to their productivity, biodiversity, and ecosystem functions (Kahmen et al., 2008; Andersen and Turner, 2013).

The N-acquisition strategies of plants are influenced by abiotic and biotic factors. The availability of soil N can affect root N uptake and substrate preference. For example, plants in boreal forests (Nordin et al., 2001), temperate forests (Zhou et al., 2019), subtropical plantations (Liu et al., 2020), and tropical forests (Liu et al., 2017) preferred to absorb ammonium where the ammonium content was higher than the nitrate level in the soil. Similarly, in areas where soil nitrate was dominant, temperate *Fagus sylvatica* (Jacob and Leuschner, 2015), *Schima superba*, and *Liquidambar formosana* (Gessler et al., 1998) preferred to absorb nitrate. Some evidence suggests that plants may prefer organic N over inorganic N, or absorb both equally, especially in low-temperature, N-restricted polar, alpine, and boreal ecosystems (Chapin et al., 1993; Näsholm et al., 1998; Öhlund and Näsholm, 2001; Simon et al., 2021). However, some studies have reported that the plant N uptake preference was in contrast to the predominant form of N in the soil. For example, *Fagus grandifolia* seedlings (Templer and Dawson, 2004), *Larix gmelinii*, and *Betula platyphylla* (Gao et al., 2020) preferred to absorb nitrate, although the content of ammonium in the soil was 2–14 times higher than that of nitrate. This indicates that the plant uptake preference for different forms of N is not only related to the availability of N in the soil but also to internal factors specific to the species. For instance, the inorganic N uptake rate of arbuscular mycorrhizal species was higher than that of ectomycorrhizal species (Liese et al., 2018). In recent years, negative correlations (Liu et al., 2020; Yi et al., 2023), positive correlations (Hong et al., 2018), and no correlation (Ma et al., 2018) have been reported regarding the linear relationship between physiological N uptake rate and the morphology of specific root length (SRL). Therefore, the root N uptake rate and preference are complex phenomena due to the combined influence of abiotic and biotic factors. In addition, the relationship between the root N uptake rate and the structural traits of the same tree species based on age is an aspect that has yet to be examined.

With the increase in tree age, the biotic and abiotic factors that affect root N absorption often change, which in turn will affect root N absorption. However, the existing research results have shown large variations in the N uptake with tree age due to differences in the study sites and tree species. Tropical *Hevea brasiliensis* aged 7–49 years showed a preference for ammonium. The ammonium uptake rate increased initially and then decreased sharply, and the glycine uptake decreased initially and then increased sharply with age (Liu et al., 2018). Subtropical *Cunninghamia lanceolata* aged 5–30 years preferred to absorb ammonium, and the ammonium uptake rates for 30- and 5-year-old trees were similar and were higher than those of 13-year-old trees (Li et al., 2016). Temperate *Pinus koraiensis* aged 14–217 years preferred ammonium, and the ammonium uptake rate decreased with tree age (Ren et al., 2021). *F. sylvatica* aged 5–130 years preferred organic glutamine and arginine, and the inorganic and glutamine uptake rates did not differ among age classes (Simon et al., 2021). Therefore, given the uncertainties in the relationship between N uptake preferences (or the uptake rates) and stand age, conducting studies in different geographic regions and forest types will contribute to a more comprehensive understanding of the N acquisition strategies during forest succession.

Root traits can reflect the underground resource strategies of tree species. Root trait variation was initially assumed to be a one-dimensional root economic spectrum with a trade-off between resource acquisition and conservation (Comas and Eissenstat, 2004; Roumet et al., 2016). The roots in nutrient- and water-rich environments were characterized by their smaller diameter, higher SRL, higher N content, lower root tissue density (RTD), shorter root life span, and lower mycorrhizal colonization rate. As such roots have high nutrient and water absorption capacity, this indicated a resource-acquisitive strategy (Roumet et al., 2006, 2016; Pinno and Wilson, 2013; McCormack et al., 2015; Fort et al., 2016; Li et al., 2017). When the water and fertilizer contents are low, plant roots show the opposite pattern, a resource-conservative strategy. However, an increasing number of studies have demonstrated that variations in root traits are multidimensional rather than reflecting a single axis related to resource economics (Kong et al., 2014; Wang et al., 2018; Kong et al., 2019; Bergmann et al., 2020; Ding et al., 2020; Weigelt et al., 2021; Yan et al., 2022). For example, Bergmann et al. (2020) analyzed the root traits of 1,810 species and proposed a two-dimensional root economic space with conservation and collaboration gradients. In addition, root exudation traits (Wen et al., 2022), root respiration (Han and Zhu, 2021), and N uptake rate (Yi et al., 2023) were integrated into the current theoretical framework of root economic space. The above studies reflect the trade-off dimensions of the root morphological, architectural, anatomical, chemical, and physiological traits across species. However, there are few studies on root economic space based on the age gradient of the same tree species.

In this study, root physiology (N uptake rate), morphology (diameter, SRL, and RTD), architecture (branching ratio and intensity), chemistry (N and C contents), and mycorrhizal colonization rate in the *L. principis-rupprechtii* chronosequence (aged 18, 27, 37, 46, and 57 years) in the Saihanba Mechanical Forest Farm in Northern China were analyzed. We aimed to verify 1) how the N uptake rates and substrate preferences of *L. principis-rupprechtii* plantations change with tree age and 2) how the economic space based on root functional traits changes along the age gradient in *L. principis-rupprechtii* plantations. Here, we hypothesized that 1) with the increase in tree age, the N uptake rate of *L. principis-rupprechtii* roots would decrease while the nitrogen preference would not vary (Simon et al., 2021; Ren et al., 2023) and 2) trees with different ages would show different N strategies, indicating two dimensions of collaborative and conservative root functional traits along the age gradient. These hypotheses were formed following the root economic space theory for multi-tree species (Bergmann et al., 2020).

## 2 Materials and methods

### 2.1 Study site and experimental design

This study was conducted at the Saihanba Mechanical Forest Farm (116°53′–117°39′ E, 41°92′–42°36′ N) in Chengde City, Hebei Province, Northern China. The elevation of the area is 1,010–1,939 m. This region has a typical semi-arid and semi-humid cold temperate continental monsoon climate, with an average annual precipitation of about 460 mm and an average annual temperature of about −1.3°C. The soil is of a gray forest type. The main tree species in this region are *L. principis-rupprechtii*, *Pinus sylvestris*, *Betula platyphylla*, *Picea asperata*, and *Populus davidiana*. The larch plantation accounts for more than 90%, with a few understory shrub species, mainly *Rosa davurica*, *Lonicera microphylla*, and *Spiraea pubescens*. There are many species of herbs in the *L. principis-rupprechtii* plantations, including *Agrimonia pilosa*, *Sanguisorba officinalis*, *Ranunculus japonicus*, *Veronica longifolia*, *Adenophora stricta*, *Carex lanceolata*, *Thalictrum petaloideum*, *Papaver nudicaule*, and *Trollius chinensis*.

In July 2021, five *L. principis-rupprechtii* plantations of different ages with similar stand conditions, good growth, and adjacent distribution were selected for the experiments. The five larch plantations were all wasteland before afforestation and had not been disturbed by human activities. The age of the trees was confirmed by the annual ring of the tree cores sampled from the growth cone. Trees with different ages were in different ontogenetic stages depending on their diameter at breast height and the height that was surveyed. **Table 1** displays the plantation characteristics. Four 20-m × 20-m plots were randomly set up for each age of the *L. principis-rupprechtii* plantations, and soils at a depth of 0–15 cm were collected with a soil drill using the five-point mixing method. In each plot, the roots of *L. principis-rupprechtii* were carefully excavated in four directions along the trunk, taking care to ensure the integrity of the fine roots. The four root samples in each plot were placed in centrifuge tubes with three ^15^N labeling solutions of ammonium, nitrate, and glycine and one controlled unlabeled solution. The concentration of each solution was 100 μmol N L^−1^ and contained a 1:1:1 nitrogen ratio of ammonium, nitrate, and glycine, with only one ^15^N form. All solutions contained 10 mg L^−1^ ampicillin to inhibit microbial activity that would degrade glycine and 200 μmol L^−1^ CaCl_2_ to maintain membrane stability (Warren and Adams, 2007). After 2 h of the labeling experiment, the roots were harvested, cleaned with 50 mmol L^−1^ KCl solution and deionized water, placed in an envelope, and brought back to the laboratory. In addition, roots were collected within 20 cm of the sampling point for isotopic labeling hydroponics, and these roots were used to determine the morphological, architectural, and mycorrhizal traits.

**Table 1:** Basic characteristics of *Larix principis-rupprechtii* plantations at different tree ages.

Tree age (years)	Longitude and latitude	Altitude (m)	Density (hm^−2^)	Diameter at breast height (cm)	Height (m)
18 (±1)	117°14′10″ E, 42°23′9″ N	1,489	3,225	11.60 ± 1.00	10.20 ± 0.58
27 (±1)	117°13′24″ E, 42°27′54″ N	1,497	3,000	13.90 ± 0.66	14.00 ± 4.67
37 (±2)	117°16′24″ E, 42°23′23″ N	1,515	2,325	21.55 ± 1.46	16.37 ± 0.42
46 (±2)	117°14′28″ E, 42°24′1″ N	1,491	1,350	24.33 ± 0.55	17.44 ± 0.34
57 (±1)	117°19′6″ E, 42°24′39″ N	1,527	1,350	31.21 ± 0.98	24.00 ± 0.31

Values are mean values with standard errors.

### 2.2 Root trait measurements

Roots were dried and ground to powder for the determination of isotope ^15^N/^14^N and total C and N contents using an isotope ratio mass spectrometer (IRMS, MAT253; Finnigan MAT, Bremen, Germany) coupled to an elemental analyzer (EA 1110; CE Instruments, Milan, Italy). Other root samples were graded, scanned on an Epson scanner at 300 dpi, dried, and weighed. The scanned images were analyzed using WinRHIZO software (Regent Instruments Inc., Quebec City, QC, Canada) to obtain the average diameter (AD), total length, total surface area, and total volume. The number of root segments in each image was obtained by counting. The calculation formulas for SRL, specific root area (SRA), RTD, root branching ratio (BR), and branching intensity (BI) were from a previous study (Liu et al., 2023). A total of 200 root segments were observed under an anatomic microscope with ×20 magnification (EZ4W; Leica, Wetzlar, Germany) to determine whether they were infected by mycorrhizal fungi based on the features of a yellow-brown or golden-brown color and a swollen appearance. The ectomycorrhizal colonization rate (ECM) was defined as the ratio of the number of root tips infected by fungi to the total number of root tips observed.

### 2.3 Soil trait measurements

Fresh soil was extracted with a 0.05 mol L^−1^ K_2_SO_4_ solution and measured using an automatic continuous flow analyzer (AA3; Bran-Luebbe, Hamburg, Germany) to obtain the NH_4_^+^ and NO_3_^−^ contents. The soil glycine concentrations were measured using high-performance liquid chromatography–tandem mass spectrometry (HPLC-MS/MS API 3200 QTRAP; CA, USA) after the derivatization of amino acids. The total C and total N were measured using an elemental analyzer (EA3000, EuroVector, Milan, Italy) after removing the inorganic C with hydrochloric acid. Total phosphorus was determined by a sodium hydroxide–molybdenum–antimony reactance colorimetric method, while total potassium was determined using flame spectrophotometry. **Table 2** displays information on the soil characteristics.

**Table 2:** Soil characteristics of *Larix principis-rupprechtii* plantations.

Age (years)	pH	Water content (%)	Organic carbon (g kg^−1^)	Total nitrogen (g kg^−1^)	N–NH_4_^+^ content (mg kg^−1^)	N–NO_3_^−^ content (mg kg^−1^)	N-glycine content (mg kg^−1^)	Total phosphorus (g kg^−1^)	Total potassium (g kg^−1^)
18	6.63 ± 0.02a	14.17 ± 0.16b	19.19 ± 0.78d	1.58 ± 0.07d	15.20 ± 0.55b	17.27 ± 1.41a	0.05 ± 0.00a	0.14 ± 0.01ab	13.88 ± 0.58b
27	6.07 ± 0.02c	22.11 ± 0.93a	24.08 ± 0.58c	2.07 ± 0.05c	14.22 ± 1.68b	18.00 ± 0.55a	0.06 ± 0.01a	0.14 ± 0.02ab	15.81 ± 0.54a
37	6.31 ± 0.02b	20.19 ± 1.33a	20.07 ± 0.65d	1.69 ± 0.23cd	29.35 ± 1.74a	13.34 ± 1.83b	0.07 ± 0.00a	0.15 ± 0.02a	12.69 ± 0.18b
46	5.83 ± 0.01e	20.79 ± 0.48a	30.57 ± 1.49b	2.94 ± 0.14b	29.97 ± 0.52a	10.54 ± 1.50b	0.06 ± 0.01a	0.11 ± 0.01b	16.02 ± 0.21a
57	5.99 ± 0.01d	21.52 ± 1.73a	36.88 ± 0.54a	3.50 ± 0.07a	33.15 ± 2.15a	12.06 ± 0.70b	0.03 ± 0.00b	0.11 ± 0.01b	16.26 ± 1.01a

Values are mean values with standard errors. Different letters represent statistical significance across tree ages (p< 0.05).

### 2.4 Calculation and statistical analyses

The N uptake rate (NUR, in micrograms N per gram of root dry weight per hour) was calculated as in the following equations (Liu et al., 2020):


(1) $$\text{NUR}=\frac{\text{N content}(\frac{\text{μg}}{\text{g}})\times \text{APE}}{\text{time}(\text{h})\times \text{atom}{\%}_{⬚}^{15}\text{N}\;\text{tracer}}$$

(2) $$\text{APE}={(}^{15}\text{N}\;\text{atom}\%\text{ excess})=A_{\text{L}}-A_{\text{CK}}$$

The atom% ^15^N values of the tracer were 10.18% for nitrate, 10.12% for ammonium, and 99.12% for glycine. *A*_L_ denotes the atom% ^15^N of labeled roots, while *A*_CK_ is the atom% ^15^N of unlabeled roots. The N uptake contribution was calculated as the uptake rate of one N form divided by the sum of three N forms.

The normal distribution of the data was verified by a non-parametric Shapiro–Wilk test in SPSS 20 (SPSS Inc., Chicago, IL, USA). Differences in the root traits among tree ages were analyzed using one-way analysis of variance (ANOVA), followed by a least significant difference (LSD) test using SPSS 20. Differences were considered significant at *p*< 0.05. Principal component analysis (PCA) of the root traits across tree ages was conducted in R v.4.0.3 (OriginLab Software Inc., Northampton, MA, USA). **Table 3** presents the abbreviations and descriptions of the root morphological, architectural, mycorrhizal, chemical, and physiological traits and soil characteristics.

**Table 3:** Abbreviations and descriptions of the root morphological, architectural, mycorrhizal, chemical, and physiological traits and soil characteristics.

Parameters		Abbreviation	Units	Description
Root morphological traits	Root diameter	AD	mm	Average root diameter
	Root tissue density	RTD	g cm^−3^	The ratio of root dry mass to root volume
	Specific root length	SRL	m g^−1^	The ratio of root length to root dry mass
	Specific root area	SRA	cm^2^ g^−1^	The ratio of root surface area to root dry mass
Root architectural traits	Branching ratio	BR	none	The ratio of the first-order root number to the second-order root number
	Branching intensity	BI	cm^−1^	The ratio of the first-order root number to the second-order root length
Mycorrhizal traits	Mycorrhizal colonization rate	ECM	%	Ectomycorrhizal colonization rate
Root chemical traits	Root carbon content	RC	%	Root carbon content
	Root nitrogen content	RN	%	Root nitrogen content
Root physiological traits	Ammonium uptake rate	UAM	µg g^−1^ h^−1^	Ammonium nitrogen uptake rate
	Nitrate uptake rate	UNT	µg g^−1^ h^−1^	Nitrate nitrogen uptake rate
	Glycine uptake rate	UGLY	µg g^−1^ h^−1^	Glycine nitrogen uptake rate
	Total nitrogen uptake rate	UTN	µg g^−1^ h^−1^	Total nitrogen uptake rate
Soil characteristics	Soil water content	SWC	%	Soil water content
	Soil total nitrogen	STN	g kg^−1^	Soil total nitrogen
	Soil total organic carbon	SOC	g kg^−1^	Soil total organic carbon
	Soil ammonium content	SAM	mg kg^−1^	Soil ammonium content
	Soil nitrate content	SNT	mg kg^−1^	Soil nitrate content
	Soil glycine content	SGLY	mg kg^−1^	Soil glycine content
	Soil total phosphorus content	STP	g kg^−1^	Soil total phosphorus content
	Soil total potassium content	STK	g kg^−1^	Soil total potassium content

## 3 Results

### 3.1 Soil characteristics with tree ages

The soil pH and total phosphorus of young (i.e., 18, 27, and 37 years old) *L. principis-rupprechtii* trees were significantly higher than those of older trees (46 and 57 years old). The organic C and total N contents in the soil of young trees were significantly lower than those of older trees (**Table 2**). The NH_4_^+^–N content in the soils of the 18- and 27-year-old trees was significantly lower, while the nitrate content was higher than that in the soils of the 37-, 46-, and 57-year-old trees (**Table 2**). The soil water content of the 18-year-old trees was the lowest, and the glycine content in the soil of 57-year-old trees was the lowest (**Table 2**).

### 3.2 Changes in the N uptake rate and preference with tree age

**Figure 1:**
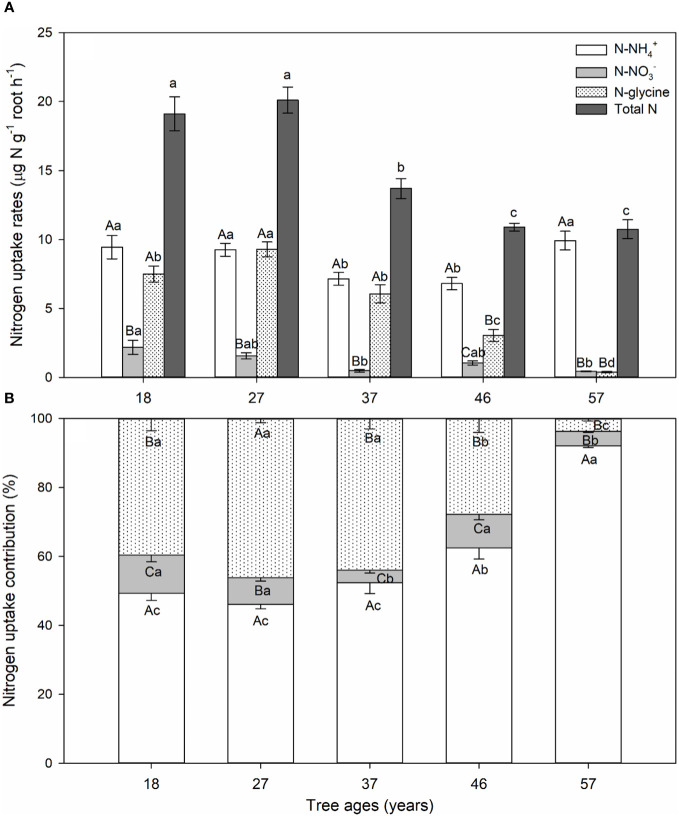
**(A)** N uptake rates. **(B)** Contributions of ammonium (NH_4_^+^), nitrate (NO_3_^−^), and glycine of *Larix principis-rupprechtii* at different tree ages. Values are presented as mean and standard error. *Different capital letters* indicate significant differences between ammonium, nitrate, and glycine uptake rates, while *different lowercase letters* indicate significant differences between tree ages (*p*< 0.05).

Tree age affected uptake rates (calculated by Equations 1, 2) for the three N sources. The ammonium uptake rate of *L. principis-rupprechtii* roots significantly decreased (aged 18–37 years) and then increased (aged 46–57 years) with increasing tree age (**Figure 1A**). The glycine uptake rate significantly increased (aged 18–27 years) and then decreased (aged 27–57 years) with increasing tree age (**Figure 1A**). The uptake rates of nitrate and total N significantly decreased with increasing tree age (aged 18–57 years).

Within the same age group, the uptake rates for the three N forms showed a significant difference. The ammonium and glycine uptake rates of young *L. principis-rupprechtii* showed no significant differences, being 4–14 times higher than the nitrate uptake rate (**Figure 1A**). The uptake rate of the 46-year-old trees was in the order of ammonium > glycine > nitrate (**Figure 1A**). The glycine and nitrate uptake rates of the 57-year-old trees showed no differences and were 22 times lower than the ammonium uptake rate (**Figure 1A**).

Across all tree ages, *L. principis-rupprechtii* preferred to absorb ammonium, followed by glycine, with the smallest proportion being nitrate (**Figure 1B**). The contribution of ammonium uptake (45%–90%) increased, while that of glycine uptake (40%–5%) decreased with increasing tree age (**Figure 1B**). The contribution of nitrate uptake decreased initially and then increased with increasing tree age, but the overall proportion was small, ranging from 4% to 11% (**Figure 1B**).

### 3.3 Root morphological, architectural, mycorrhizal, and chemical traits with tree ages

**Figure 2:**
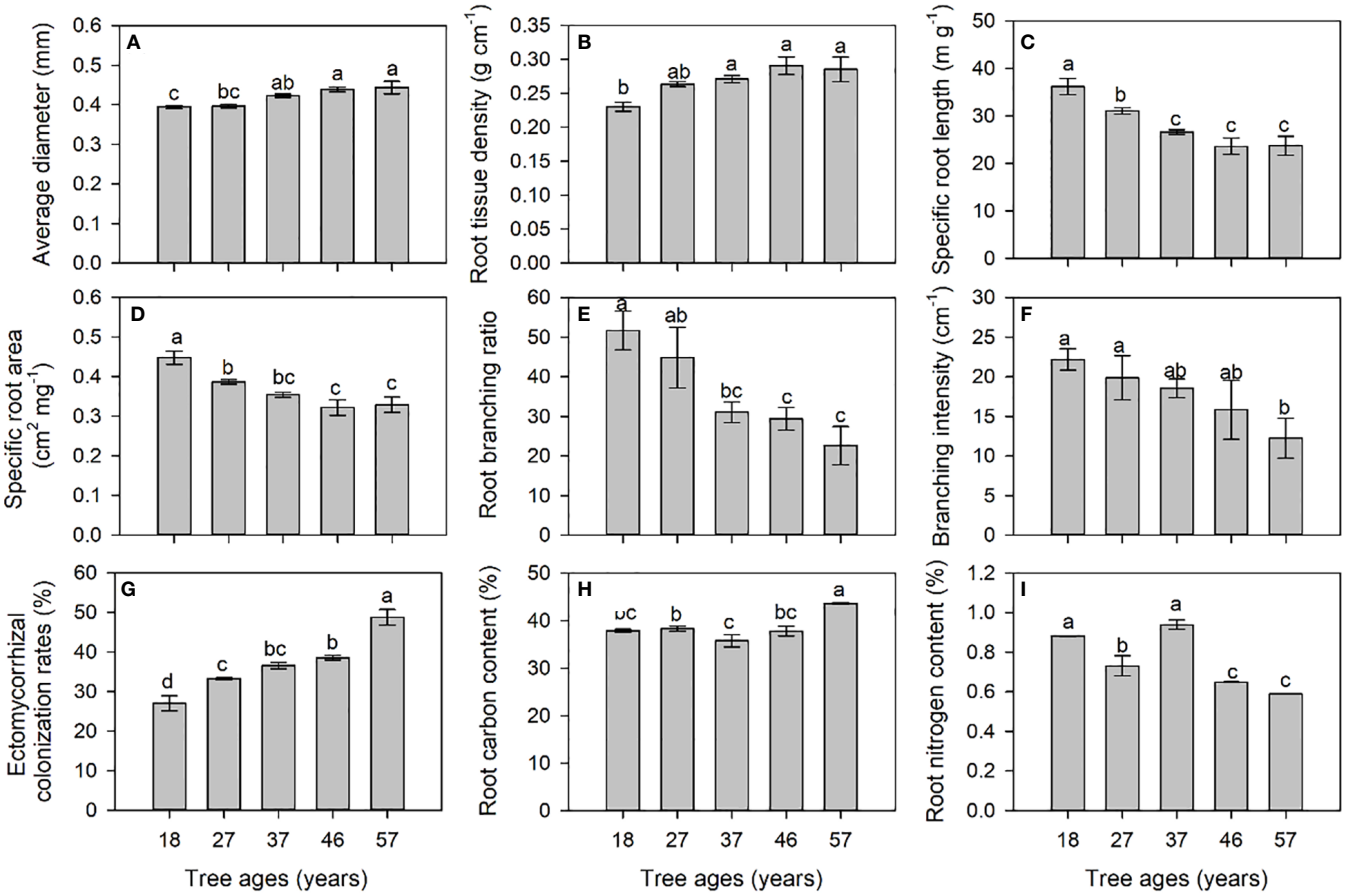
Morphological [average diameter **(A)**, root tissue density **(B)**, specific root length **(C)**, and specific root area **(D)**], architectural [root branching ratio **(E)** and branching intensity **(F)**], mycorrhizal colonization **(G)**, and chemical [root carbon **(H)** and root nitrogen **(I)**] traits of roots at different tree ages. *Different small letters* indicate significant differences between tree ages at *p*< 0.05.

The diameter, RTD, and ECM of the roots of *L. principis-rupprechtii* increased significantly with the increase in tree age (**Figures 2A, B, G**). The SRL, SRA, BR, and BI decreased significantly with increasing tree age (**Figures 2C–F**). The root carbon content (RC) of the 57-year-old trees was significantly higher than those of the other four ages, and the root nitrogen (RN) content of young trees was significantly higher than that of older trees (**Figures 2H, I**).

### 3.4 Relationships among root traits and soil characteristics

**Figure 3:**
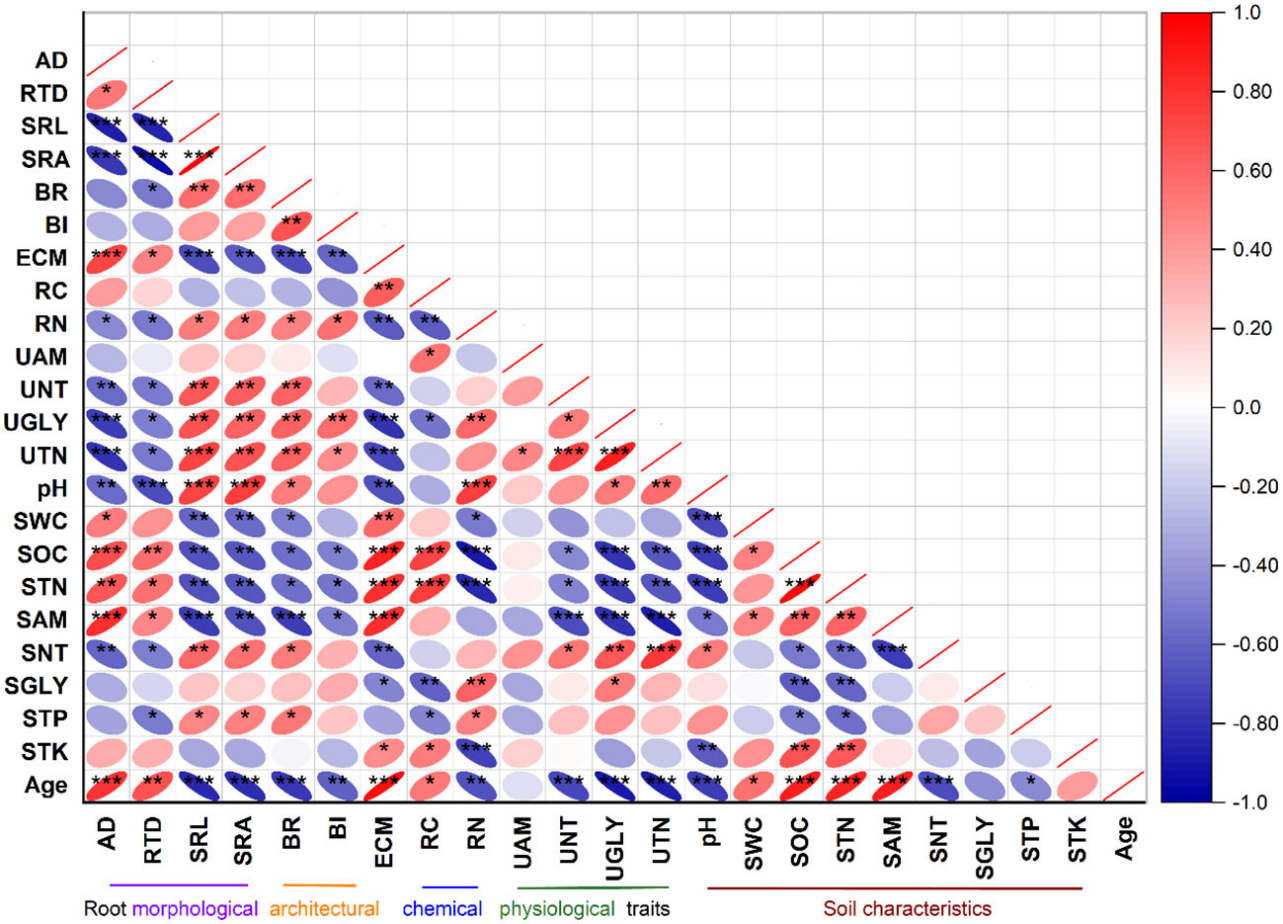
Pearson’s correlation analysis between root traits and soil characteristics. *Asterisks* indicate significance at *p< 0.05, **p< 0.01, and ***p< 0.001 levels. Abbreviations and descriptions are listed in **Table 3**.

The glycine, nitrate, and total N uptake rates were positively correlated with the soil glycine and nitrate contents but negatively associated with the soil organic C, total N, and ammonium contents (**Figure 3**). The glycine, nitrate, and total N uptake rates were positively correlated with SRL, SRA, BR, BI, and RN but negatively correlated with the AD, RTD, ECM, and RC of roots. The ammonium uptake rate was weakly correlated with the soil properties and root traits (**Figure 3**).

Soil pH was positively correlated with root traits such as SRL, SRA, BR, and RN (*p*< 0.05), but negatively associated with the AD, RTD, and ECM of roots (*p*< 0.05) (**Figure 3**). The soil water content and the organic C, total N, and ammonium contents displayed significant positive correlations with the AD, RTD, and ECM of roots but were negatively correlated with the SRL, SRA, BR, BI, and RN of roots (**Figure 3**). The soil nitrate content exhibited significant positive relationships with SRL, SRA, and BR but was negatively correlated with the root diameter (AD), RTD, and ECM (*p*< 0.05) of roots (**Figure 3**).

**Figure 4:**
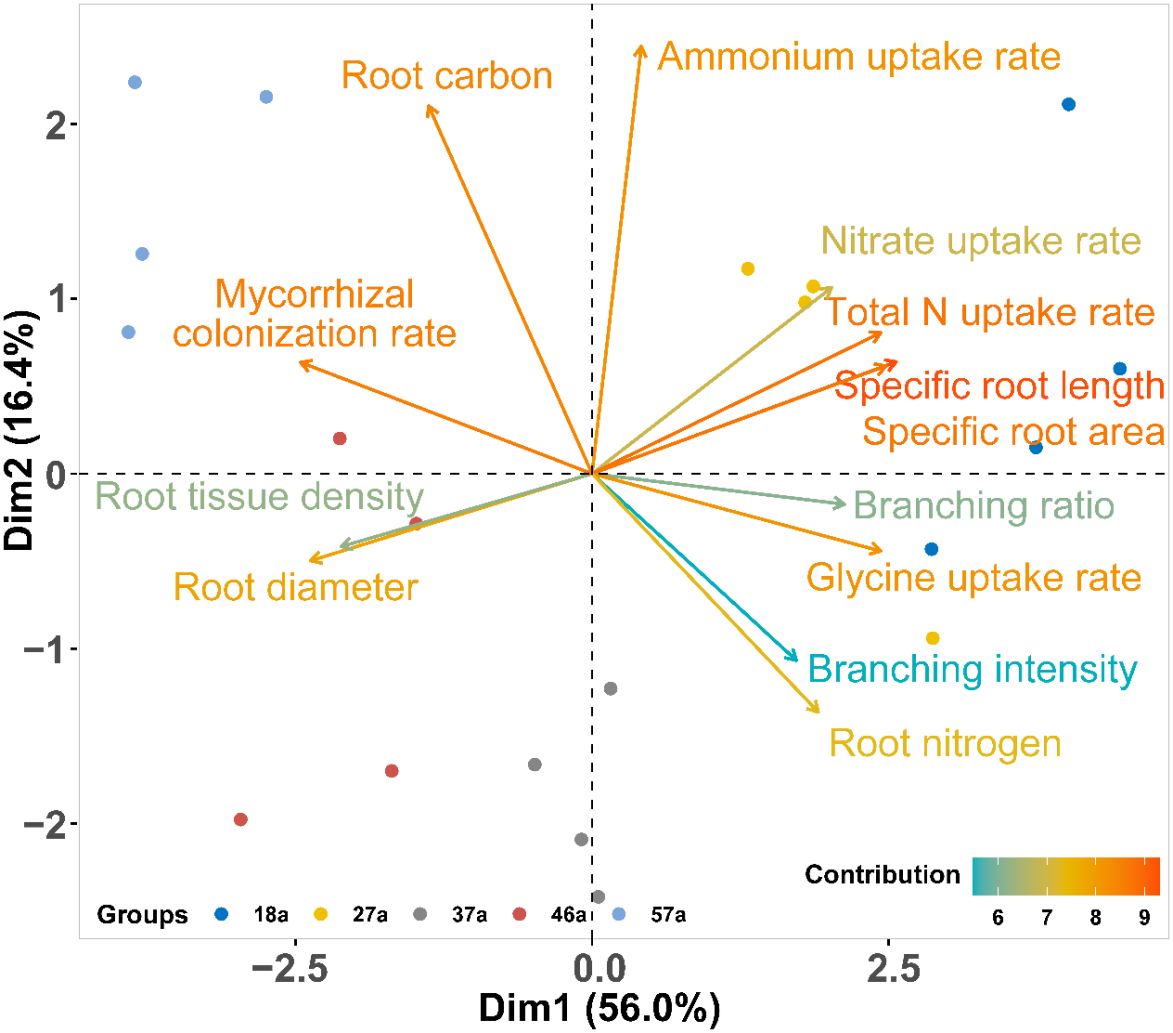
Principal component analysis (PCA) for root traits across tree ages. The *colors of the lines* represent the total contribution of each variable to the first and second principal components.

**Figure 5:**
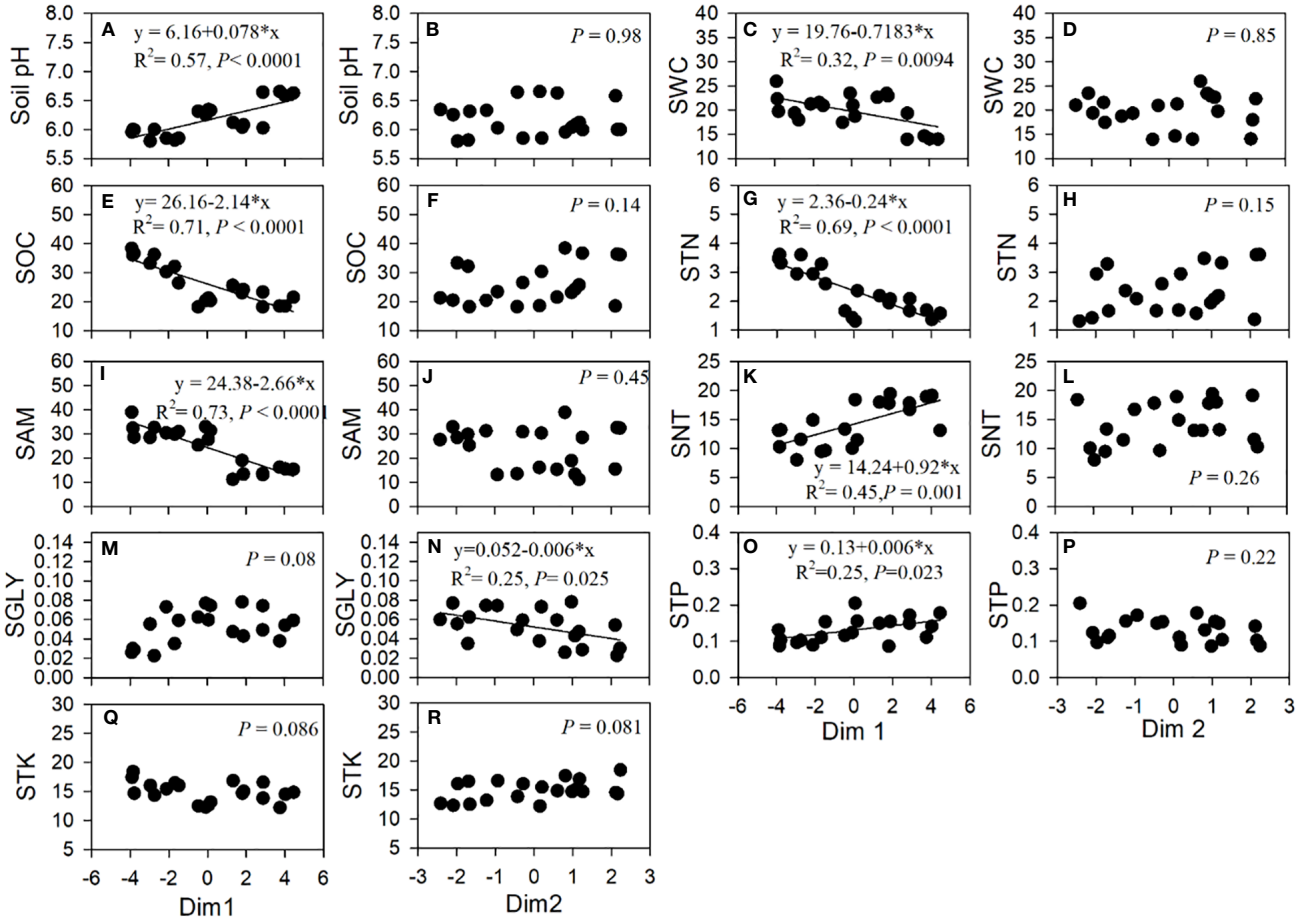
Linear relationships of the axes in the principal component analysis and soil factors. *SWC*, soil water content; *SOC*, soil organic carbon; *STN*, soil total nitrogen; *SAM*, soil ammonium content; *SNT*, soil nitrate content; *SGLY*, soil glycine content; *STP*, soil total phosphorus content; *STK*, soil total potassium content.

The PCA of the correlations of 13 root traits revealed that 72.4% of the total variation across tree age was reflected in the first two axes, of which 56.0% was attributed to the first axis (Dim1) (**Figure 4**). Dim1 was positively dominated by the total N, glycine, and nitrate uptake rates and the SRL, SRA, and BR, but negatively dominated by AD, RTD, and the ECM. Dim2 explained 16.4% of the variation and was positively associated with the ammonium uptake rate and root carbon but negatively correlated with RN and BI (**Figure 4**). The first axis was positively correlated with soil pH and the nitrate and total phosphorus contents, but negatively associated with the soil water content and the organic C, total N, and ammonium contents. The second axis was negatively correlated with soil glycine (**Figure 5**).

## 4 Discussion

In the long-term evolutionary process, plants have formed absorption mechanisms for different forms of nitrogen, generally showing a preference for a specific N form (Bueno et al., 2019). In this study, *L. principis-rupprechtii* preferred to absorb ammonium and glycine, and the contribution of ammonium increased while that of glycine decreased with tree age. Our study showed that the preference for ammonium uptake did not change with age, supporting our first hypothesis. This is consistent with previous studies demonstrating that trees (e.g., *C. lanceolata*, *H. brasiliensis*, and *P. koraiensis*) of different ages showed a preference for ammonium (Li et al., 2016; Liu et al., 2018; Ren et al., 2021). Firstly, the nitrate in plants needs to be converted into ammonium, then glutamate, and then further utilized by plants (Wang and Macko, 2011). Therefore, the energy required for plants to utilize ammonium is less than that for nitrate; this may be one of the reasons for plants preferring ammonium. Another reason could be that ammonium is abundant in soils, and the content of ammonium increased with increasing forest age in this study (**Table 2**). Other studies have found that tree species prefer to absorb ammonium, consistent with the predominance of ammonium in soils (Finzi and Berthrong, 2005; Liu et al., 2017; Zhou et al., 2019). Moreover, Zhang et al. (2018) found that the N uptake preference of *P. asperata* in the Tibetan Plateau changed from nitrate to ammonium nitrogen with increasing age and speculated that this was related to the change in the dominant N form in the soil.

The ammonium uptake rate of *L. principis-rupprechtii* roots significantly decreased and then increased, while the glycine and nitrate uptake rates decreased with increasing tree age, partly supporting our first hypothesis. This is in contrast to previous studies. Liu et al. (2018) found that the ammonium uptake rate of *H. brasiliensis* increased initially and then decreased sharply, while the glycine uptake decreased initially and then increased sharply with age. The ammonium uptake rate of *P. koraiensis* decreased with tree age (Ren et al., 2021), while that of *F. sylvatica* did not differ (Simon et al., 2021) with tree age. The reason for the contradictory findings of nitrogen absorption with age may be related to the differences in tree species and the availability of soil nitrogen. In this study, the nitrate and glycine uptake rates were positively linearly correlated with the nitrate and glycine contents in soils across the age gradient. Another reason for the changes in N uptake by plants with age may be related to the root traits of the species. The contribution of ammonium to the N uptake increased with tree age as a result of the decreased nitrate and glycine uptake in older *L. principis-rupprechtii*. Compared to nitrate, the diffusion rate of ammonium ions in the soil is much lower (Barber, 1995), suggesting that, in the case of ammonium, the roots should grow closer (and show increased branching) to the N source for more efficient uptake. Moreover, localized ammonium increases lateral root branching, while nitrate induces lateral root elongation (Remans et al., 2006; Lima et al., 2010). In our study, with the increase in forest age, the decrease in soil nitrate content was accompanied by a decrease in SRA. The decreased nitrate uptake in older trees could be caused by the ECM increasing with tree age (**Figure 2**), with the ability of the colonized root tips to take up nutrients directly disappearing as a result.

In this study, the total N uptake rate decreased significantly with increasing tree age, indicating that the nitrogen demand of older trees was relatively reduced compared to that of younger trees. This is consistent with previous studies showing that the root total N uptake rate of rubber trees and Korean pine decreased gradually with the increase in tree age (Liu et al., 2018; Ren et al., 2021). One possible reason is that younger trees grow at a faster rate and have higher nutrient requirements for adding biomass than older trees (Borchert, 1975; Ryan et al., 1997). As trees age, their growth rates gradually reach a maximum, and the available N in the soil tends to decrease (Gower et al., 1996; Tang et al., 2014). A second reason for the decrease in the N uptake rate of roots with increasing tree age may be related to leaf N resorption. Significant positive correlations between leaf N resorption efficiency and tree age have been observed in *L. principis-rupprechtii* plantations (Sun et al., 2016). As a mature forest tree, the older larch is a stronger N reservoir and has a greater N retention capacity than the younger tree (Simon et al., 2011; Sun et al., 2016); therefore, its growth may depend more on the nutrient recovery of its own organs than on the available nutrients in the soil. Although the soil types and stand conditions of our five age plots were similar, we still cannot rule out the effect of original soil differences on the N uptake rate.

The SRL and specific root surface area of *L. principis-rupprechtii* decreased with the increase in tree age as a result of increased root diameter and tissue density, similar to previous results. For example, Rosenvald et al. (2013) confirmed that the diameter and RTD of the first-order roots of *Betula pendula* gradually increased, while the SRL and SRA gradually decreased with the increase in forest age, ranging from 3 to 60 years. Moreover, for fine roots less than 2 mm in diameter, younger trees having higher values of SRL and SRA than older trees were reported in *Cryptomeria japonica* (Fujimaki et al., 2007), *P. sylvestris* (Jagodzinski and Kalucka, 2010), *B. pendula* (Kalliokoski et al., 2010), *F. sylvatica*, *Quercus robur*, *Alnus glutinosa* (Jagodzinski et al., 2016), *Fraxinus velutina* (Li et al., 2020), and *Fraxinus mandshurica* (Li et al., 2021). Considering the lower RC in younger *L. principis-rupprechtii*, the higher values of SRA at a young age indicate rapid growth and low construction costs, constituting a cost-saving method for acquiring soil resources. Compared to older trees, younger trees may need to develop more effective root systems to cope with survival pressures such as limited sunlight and soil resources. In this study, the root BR (the ratio of the tips of first-order roots to second-order roots) and BI (tips of first-order roots per length of second-order roots) were higher in young larch than in older larch. Previous studies have found that the root branching frequency (measured as tips per root dry mass) was higher in younger trees (Rosenvald et al., 2013). However, Børja et al. (2008) found that stand age had no effect on the root branching frequency of Norway spruce plantations. Normally, roots with higher SRL, SRA, and branches, but lower RTD and diameter, indicate a greater absorption capacity for soil resources (Wang et al., 2016; Weemstra et al., 2016). In our study, roots with lower diameter and RTD and higher SRL, SRA, BR, and BI exhibited higher nitrate, glycine, and total N uptake rates. Therefore, younger trees tended to develop roots with more morphologically and physiologically efficient nutrient uptake capacity to compensate for the smaller size of the root system.

**Figure 6:**
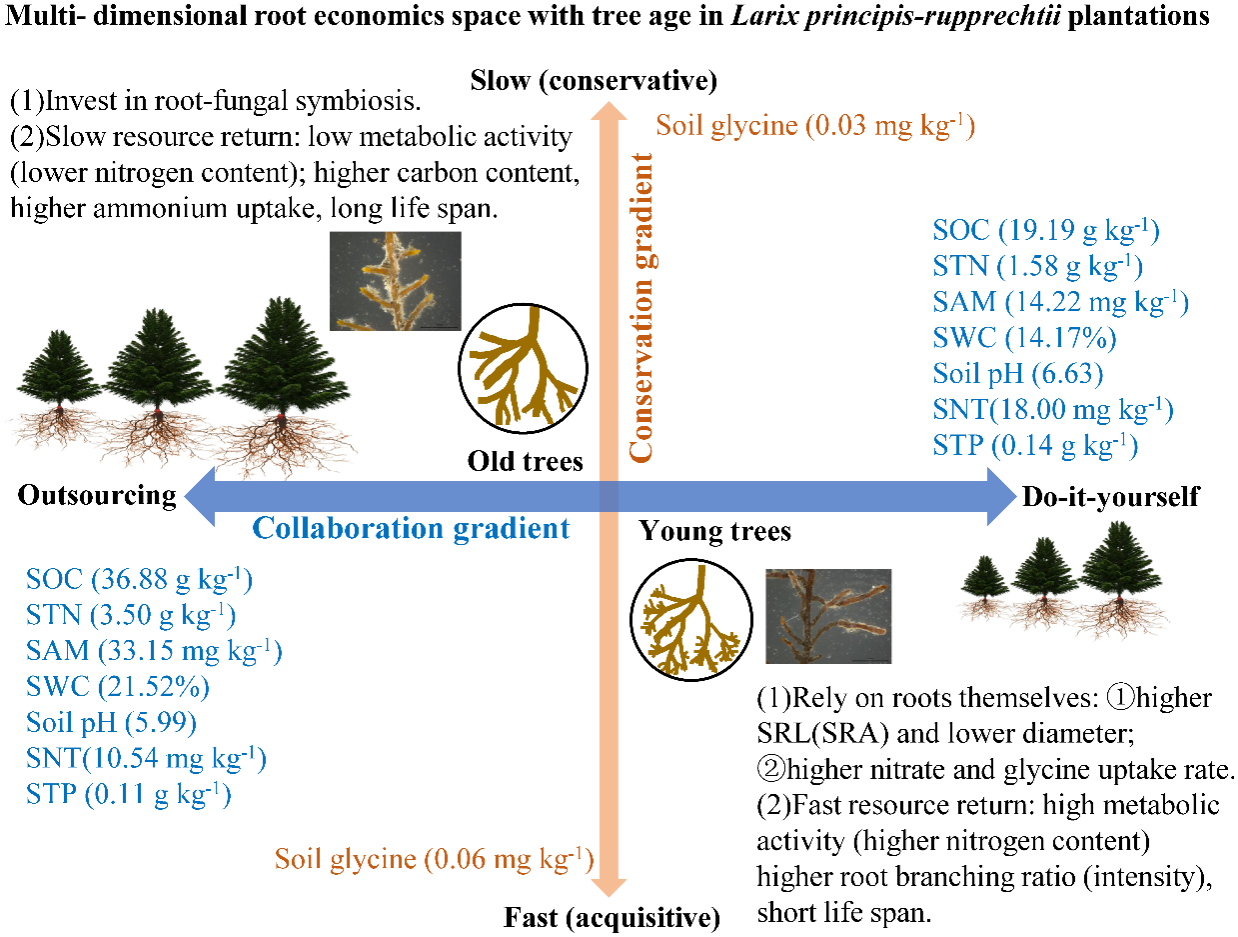
Root nitrogen acquisition strategies with tree age based on root economic space. *SWC*, soil water content; *SOC*, soil organic carbon; *STN*, soil total nitrogen; *SAM*, soil ammonium content; *SNT*, soil nitrate content; *STP*, soil total phosphorus content; *SRL*, specific root length; *SRA*, specific root area.

Mycorrhizae and the physiological N uptake rates are important factors in root resource acquisition, but they have seldom been investigated from the standpoint of the theory of root economic space under intraspecies changes across tree ages. The PCA showed that the root functional traits (i.e., morphology, architecture, chemistry, mycorrhizal symbioses, and physiology) loaded onto two predominant axes comprising “collaboration” and “conservation” gradients in belowground resource acquisition in the *L. principis-rupprechtii* chronosequence (**Figures 4**, **6**). This result supported our second hypothesis that trees with different ages show different N strategies, appearing to shift from an acquisitive strategy to a conservative strategy associated with increasing tree age. Along the collaboration gradient, younger *L. principis-rupprechtii* tended to invest more carbon to develop the root system itself for resource exploration—a “do-it-yourself” strategy (Bergmann et al., 2020) that involved features such as increased SRL and SRA, decreased RTD and diameter (morphology), and increased uptake rates of nitrate and glycine (physiology). Conversely, older *L. principis-rupprechtii* invested more carbon in acquiring mycorrhizal partners—an “outsourcing” strategy (Bergmann et al., 2020). This variation in root functional traits with age on the “collaboration” axis is consistent with the study of Ren et al. (2023) confirming a “collaboration” gradient of root economic space identified for *P. koraiensis*, *Picea koraiensis*, and *Abies nephrolepis* in three age classes. In contrast, the “conservative” axis of root chemical and architectural traits was orthogonal to the “collaboration” axis, indicating the multidimensional aspect of root economic space. The conservative–acquisitive gradient represents the fast–slow trade-offs between traits associated with high metabolic activity (e.g., root nitrogen and respiration rate) and root construction costs (e.g., root carbon and RTD) (McCormack et al., 2015; Roumet et al., 2016; Freschet and Roumet, 2017). The relatively efficient roots associated with higher N content and BI and lower C content of younger *L. principis-rupprechtii* indicated a fast, resource-acquisitive strategy. Older trees with thicker roots exhibited a slower, resource-conserving strategy.

## 5 Conclusion

The uptake rates of nitrate, glycine, and total N of *L. principis-rupprechtii* decreased with increasing tree age, while the uptake rates of ammonium decreased initially and then increased with tree age. Across all tree ages, *L. principis-rupprechtii* preferred to absorb ammonium (45%–90%), followed by glycine (5%–40%) and nitrate (4%–11%). The glycine and nitrate uptake rates were positively correlated with the soil glycine and nitrate contents. The AD, tissue density, and ECM of the roots increased, while the SRL, SRA, BR, and BI decreased with increasing tree age in *L. principis-rupprechtii* plantations. We found that the strategies of resource acquisition along the age gradient of the same tree species showed a two-dimensional root economic space, and the root functional traits varied along the conservation and collaboration gradients. In *L. principis-rupprechtii* plantations, the root resource acquisition strategy appears to shift from an acquisitive strategy to a conservative strategy associated with increasing tree age. Along the collaboration gradient, younger trees relied more on their own root morphology and physiology to acquire soil resources—a “do-it-yourself” strategy, as reflected by the increased SRL and SRA and the increased uptake rate. Conversely, older *L. principis-rupprechtii* depended more on mycorrhizal partners—an “outsourcing” strategy.

## Data availability statement

The original contributions presented in the study are included in the article/supplementary material. Further inquiries can be directed to the corresponding authors.

## Author contributions

QL: Conceptualization, Formal analysis, Funding acquisition, Investigation, Methodology, Writing – original draft, Writing – review & editing. YXC: Investigation, Writing – review & editing. YMC: Conceptualization, Writing – original draft, Writing – review & editing.
